# Preliminary validation of the Mental Health Test in a psychiatric sample

**DOI:** 10.1038/s41598-024-54537-4

**Published:** 2024-02-18

**Authors:** Virág Zábó, Dávid Erát, Xenia Gonda, Judit Harangozó, Máté Iváncsics, Ágnes Vincze, Judit Farkas, Gábor Balogh, Attila Oláh, Szabolcs Kéri, György Purebl, András Vargha

**Affiliations:** 1 Doctoral School of Psychology, ELTE Eötvös Loránd University, Budapest, Hungary; 2 Institute of Psychology, ELTE Eötvös Loránd University, Budapest, Hungary; 3 Institute of Behavioural Sciences, Semmelweis University, Budapest, Hungary; 4 Department of Sociology, Institute of Social and Media Studies, University of Pécs, Pécs, Hungary; 5 Department of Psychiatry and Psychotherapy, Semmelweis University, Budapest, Hungary; 6 NAP3.0-SE Neuropsychopharmacology Research Group, Hungarian Brain Research Program, Semmelweis University, Budapest, Hungary; 7 Community Psychiatry Centre, Semmelweis University – Awakenings Foundation, Budapest, Hungary; 8 National Institute of Mental Health, Neurology, and Neurosurgery, Nyírő Gyula Hospital, Budapest, Hungary; 9 Department of Cognitive Science, Budapest University of Technology and Economics, Budapest, Hungary; 10 Person- and Family-Oriented Health Science Research Group, Faculty of Humanities and Social Sciences, Institute of Psychology, Károli Gáspár University of the Reformed Church in Hungary, Budapest, Hungary

**Keywords:** Mental health, Mental disorders, Mental Health Test, Maintainable Positive Mental Health Theory, Mental health measures, Psychology, Human behaviour, Psychiatric disorders

## Abstract

To assist psychiatrists and clinical psychologists to assess their patients’ psychological immune competence-based capacities and resources, depending on the mental health disorder diagnosis and the severity of the symptoms, the present study examined the psychometric properties of the Mental Health Test in a psychiatric sample. The research was carried out in four Hungarian healthcare facilities using a cross-sectional design. A total of 331 patients (140 male, 188 female, and 3 who preferred not to disclose their gender) completed the Mental Health Test, six well-being and mental health measures, and the Symptom Checklist-90. Psychiatrists and clinical psychologists reported the mental disorder status of each participant. Confirmatory factor analysis showed a good fit of the five-factor model to the data for the clinical version of the Mental Health Test (CFI = 0.972, RMSEA = 0.034). High internal consistency coefficients (α: 0.70–0.84; ω: 0.71–0.85) and excellent external and content validity were reported. The test is not sensitive to sociodemographic indicators but is sensitive to the correlates of well-being and to the symptoms of different types of mental disorders. Our preliminary findings suggest that the Mental Health Test is a suitable measure for assessing mental health capacities and resources in psychiatric samples.

## Introduction

Just as we have an immune system to defend against harmful biological agents, our minds also need psychological immunity to remain resilient to the stressors we encounter in everyday life^1,2^. Previous models of mental health (or well-being more broadly) have explored various conceptualizations, such as (1) multidimensional well-being^3,4^, (2) mirror opposite to the symptoms of mental disorders^5,6^, (3) psychosocial flourishing^7^, (4) “hedo-eudemonic” well-being^8^, (5) classical models of mental health^9^, (6) balanced models of mental health^10^, and (7) the sum of the components of well-being^11^. These concepts have focused on the components of well-being, and they have not fully captured all aspects of mental health as defined by the World Health Organization (WHO)^12^ and classical theories of mental health. Therefore, to fully represent mental health, measurement tools should go beyond operationalisations that define the concept in terms of observable characteristics of well-being or as a mirror opposite of mental disorders.

The Maintainable Positive Mental Health Theory (MPMHT)^13,14^ is the first attempt to treat all theory-based and empirically identified components of well-being as the set of features of mental health that reflect the presence and proper functioning of the psychological capacities needed to maintain and promote a positive psychological status and positive mental health. The suggested definition of positive mental health is a high level of global well-being, which goes together with psychological, social, and spiritual well-functioning, psychological resilience, efficient creative executive functioning, and coping and savoring capacities, all of which are pillars that enable flourishing. Unlike the effects of vaccines, these psychological skills and competencies are deeply ingrained and enduring, developed through time. These pillars of positive mental health can ensure flourishing even when the individual faces negative events, challenges, mental and physical health issues, or possible losses.

To the best of our knowledge, the Mental Health Test (MHT)^14^, (see Supplementary Table 7 and Table 8) which operationalises the MPMHT, is the first measure that offers a comprehensive, five-dimensional framework designed to cover the wide spectrum of psychological resources connected to mental health, equally including individuals who have mental disorders.

The first factor is *Global Well-being*, which integrates existing well-being theories and encompasses multi-component subjective well-being in emotional, psychological, social, and spiritual domains of life^15–17^. *Savoring* is the second factor, referring to the ability to mentally relive joyful memories and experiences, generating mental well-being and extending it to future events^18^. Savoring is an indispensable component of MPMHT as it enhances attainment and sustainability of positive mental health^19^. The third factor, *Creative and Executive Efficiency*, facilitates individuals in dealing with obstacles and hurdles by utilizing their competencies in both personal and social problem-solving^15,20^. The fourth factor is *Self-regulation*-the capability to regulate and control temperament, emotions, and negative states while persisting in achieving a goal. This ability plays a crucial role in mental health and represents one of the most adaptive aspects of human behaviour^21–23^. Finally, *Resilience* is the fifth factor, which refers to an individual’s psychological ability to mobilise their resources and maintain positive mental health when confronted with unexpected, stressful situations. The higher the level of resilience, the quicker the individual can regain their equilibrium from such circumstances^24–26^. The MHT score provides a broad overview of the level of the patient’s mental health capacities. Once the patient’s accessible mental health capacities have been identified, these can be integrated into the recovery phase to improve effectiveness, while more adaptive intervention methods can be used in everyday clinical practice (e.g., psychotherapy). The study by Zábó et al.^14^, which presents the validation of the measurement on sine morbo samples, provides a more detailed presentation of the advantages of the new concept and measurement.

The present study aims to examine: (1) the reliability and validity of the MHT in a Hungarian adult psychiatric sample; (2) the relationship between the MHT and sociodemographic indicators; (3) the associations between MHT scores and a wide variety of indicators related to organic symptoms and physical health; (4) the relationship between MHT scores and mental disorders and symptoms; (5) the associations between the MHT and its pillars and the type of mental disorder, the severity of the principal diagnosis, psychotherapy during care, pharmacotherapy during care, and the number of self-reported mental disorders, after adjusting for socioeconomic factors; and (6) the relationship between mental health and combinations of psychotherapy and pharmacotherapy.

## Methods

### Sample and procedure

A cross-sectional, case-control design was employed to measure mental health and mental disorders. Data were gathered between 22 April 2022 and 2 February 2023 from four healthcare facilities under the following conditions. In the Department of Psychiatry and Psychotherapy of Semmelweis University, data collection took place among inpatients after their medication had been adjusted (1.5 to 2 weeks after admission). Patients at the Community Psychiatry Centre of Semmelweis University filled out a self-administered questionnaire during their first medical examination. Data from outpatients at the Psychosomatic Center of the Institute of Behavioural Sciences of Semmelweis University were gathered during the patients’ third therapy session. Data were collected from inpatients at the National Institute of Mental Health, Neurology, and Neurosurgery at the Nyírő Gyula Hospital after the adjustment of their medication (1.5 to 2 weeks after admission), and from outpatients during their third therapy session. Ethical approval (ethical permission number: IV/2423-3/2022/EKU) was obtained from the national Medical Research Council. The study was conducted in accordance with the relevant guidelines and regulations. Informed consent was obtained from all the participants. The sample received the information statement, consent form, and questionnaire in paper format. In a separate document, the patient’s psychiatrist or clinical psychologist provided information about the diagnosis of the patient’s mental disorder(s), the severity of the presenting symptoms, and the patient’s pharmacotherapy. The inclusion criteria were: (1) age: 18–80 years; (2) voluntary participation; and (3) diagnosis with (a) mental disorder(s). The exclusion criterion was a condition that impaired cognitive function and prevented the completion of the questionnaire. A total of 331 patients (140 male, 188 female, and 3 who preferred not to disclose their gender), aged M = 42.5 (SD = 15.9), participated in the study. Further sociodemographic indicators of the sample are presented in Table 1.

**Table 1:** Sociodemographic characteristics of the participants (*n* = 331).

Characteristic	N	Percentage/mean (SD)
Age	331	42.5 (15.9)
Gender		
Male	140	42.3
Female	188	56.8
Did not disclose gender	3	0.9
Main diagnosis		
Addictive disorders	56	16.9
Affective disorders	45	13.6
Anxiety and somatization disorders	29	8.8
Eating disorders	11	3.3
Neurocognitive disorders and intellectual disability	4	1.2
Personality disorders	39	11.8
Psychotic disorders	59	17.8
Unipolar depression	44	13.3
Employment		
Employed	160	48.3
Unemployed	162	48.9
Marital status		
Not in a relationship	181	54.7
In a relationship	149	45.0
Religiousness		
Religious	59	17.8
Religious regardless of denomination	95	28.7
Not religious	175	45.0
Education		
Elementary or lower	46	13.9
Secondary	154	46.5
Tertiary	126	38.1
Subsamples		
Community Psychiatry Center, Semmelweis University	19	5.7
National Institute of Mental Health, Neurology, and Neurosurgery at the Nyírő Gyula Hospital, Psychiatric Ward D: inpatients	49	14.8
National Institute of Mental Health, Neurology, and Neurosurgery at the Nyírő Gyula Hospital, Psychiatric Ward D: outpatients	5	1.5
National Institute of Mental Health, Neurology, and Neurosurgery at the Nyírő Gyula Hospital, Department of Addictology	33	10.0
National Institute of Mental Health, Neurology, and Neurosurgery at the Nyírő Gyula Hospital, Acute Psychiatric Ward A	56	16.9
Psychosomatic Center of the Institute of Behavioural Sciences, Semmelweis University	33	10.0
Department of Psychiatry and Psychotherapy of Semmelweis University	136	41.1

Due to missing cases, the number of participants does not add up to 331 for all indicators.

### Measures

Participants received a printed, 226-item self-report questionnaire. Fourteen questions referred to sociodemographic data. Twenty-seven questions measured general mental and physical health with single items. One question assessed the proportion of the respondent’s recent positive experiences. Participants reported: (1) if they thought they had (a) mental disorder(s); (2) what symptoms they experienced and how intensely; and (3) whether they had ever been diagnosed with a mental disorder. The instruments used were the Mental Health Test^13,14^; the Global Well-being Scale^15^; the PERMA-profiler^27^; the Psychological Well-being Scale^3^; the Satisfaction with Life Scale^28^; the Positivity Scale^6^; and the Symptom Checklist-90, revised^29^. Details of the measures are given in Supplementary Table 1. Each respondent’s psychiatrist or clinical psychologist was asked to provide a paper-based report on the patient, including: (1) the name of the patient’s mental disorder(s) according to DSM-5^30^ or ICD^31^, depending on the institution’s protocol; (2) the severity of the symptoms; and (3) the patient’s pharmacotherapy.

### Statistical processing

Regarding our analytical strategy, we rely on several commonly employed methods. First, for analyses related to structural validity, we employed confirmatory factor analysis (CFA) consistent with the analyses described in the original article introducing the MHT^14^. We chose a robust method for model fitting (maximum likelihood mean variance, MLMV) in CFA, which provides a good alternative to the traditional ML method requiring multidimensional normality^32^. Furthermore, we calculated two reliability measures for the proposed subscales, namely, Cronbach’s alpha and McDonald’s Omega^33^. Second, to assess intercorrelations between the subscales or between the scales and other variables, we calculated both Pearson’s product-moment and Spearman’s rank-based correlation coefficients. If normality is violated (noted under the tables) which results in large differences between the two measures^34^ then Spearman coefficients are used, otherwise all results are based on the former. To examine differences between sociodemographic groups, we employ robust t-tests (which account for unequal variances, see the study by Derrick, Toher and White^35^ if normality is confirmed (noted under tables), and one-way ANOVAs^36^. Finally, we conclude our examinations with multivariate ordinary least squares linear regression models^37^, where the dependent variables are the MHT scales. We check for the violation of the OLS linear regression assumptions using visual methods^37^. All calculations and results are available from the authors.

## Results

### Structural validity

Confirmatory factor analysis (CFA) was performed with R and ROP-R^38^. The values obtained indicated a good fit for all indicators. The most important results are summarized in Table 2.

**Table 2:** The main model fit indices in confirmatory factor analysis of the five-factor model of the MHT on a clinical sample.

Chi-square	AIC, BIC	RMSEA	CI._90_ (RMSEA)	pClose	CFI	TLI	SRMR
149.19* (df = 108)	17,998.2, 18,233.9	0.034	[0.019–0.047]	0.984	0.972	0.965	0.052

*p < 0.005.

Table 3 shows the alpha and omega values with 95% confidence intervals, with all subscales showing acceptable internal consistency.

**Table 3:** Measures of reliability for the subscales of the MHT.

Subscale	Number of items/subscales	Cronbach’s α	McDonald’s ω
Global well-being	3	0.84 (0.79–0.87)	0.84 (0.79–0.87)
Savoring	3	0.76 (0.71–0.81)	0.76 (0.71–0.81)
Creative and executive efficiency	5	0.84 (0.81–0.87)	0.85 (0.82–0.88)
Self-regulation	3	0.70 (0.64–0.76)	0.71 (0.64–0.76)
Psychological resilience	3	0.79 (0.73–0.83)	0.81 (0.76–0.84)
MHT score	5	0.74 (0.70–0.78)	0.77 (0.72–0.80)

N = 331. Parentheses show bootstrapped confidence intervals (95%, 10,000 runs). MHT score is the average of the subscales.

The intercorrelations of the subscales (see Table 4) indicate that in the clinical sample, the MHT subscales have a strong positive relationship with very large effect size (*r* = 0.43–0.61, *p* < 0.001), with two weaker but still significant correlations with small and medium effect sizes (*r* = 0.19 and 0.26, *p* < 0.001)^39^. In two instances, the scores for the subscales were not associated with each other: our data suggest that savoring and creative and executive efficiency are not related to self-regulation in the clinical sample. Descriptive statistics of the MHT subscales are shown in Supplementary Table 4, descriptive statistics of the single items of the MHT are shown in Supplementary Table 5. Density plots for MHT subscales are shown in Supplementary Figure 1.

**Table 4:** Intercorrelations of the MHT subscales.

Subscale	Global well-being	Savoring	Creative and executive efficiency	Self-regulation	Psychological resilience	MHT score
Global well-being	–	0.59* (0.52–0.66)	0.43* (0.34–0.51)	0.19* (0.09 to 0.29)	0.61* (0.53–0.67)	0.81* (0.77–0.85)
Savoring	–	–	0.46* (0.38–0.55)	− 0.01 (− 0.11 to 0.09)	0.49* (0.41–0.57)	0.73* (0.68–0.78)
Creative and executive efficiency	–	–	–	0.07 (− 0.04 to 0.18)	0.50* (0.42–0.58)	0.69* (0.64–0.75)
Self-regulation	–	–	–	–	0.26* (0.16–0.36)	0.44* (0.34–0.52)
Psychological resilience	–	–	–	–	–	0.82* (0.78–0.85)
MHT score	–	–	–	–	–	–

N = 331. Parentheses show confidence intervals (95%) of the presented Pearson correlation coefficients. We compared our results to robust rank-based correlation coefficients (Spearman), with a maximum difference of 0.02 in the absolute strength of correlation (results available from the authors). MHT score is the average of the subscales.

*p < 0.001.

### External and content validity

For the analysis of content validity, we estimated the (Pearson) correlation between the five MHT subscales and the previously described instruments (see Table 5; descriptive statitistics of the other instruments used in the study are shown in Supplementary Table 2; measures of reliability for the other instruments used are shown in Supplementary Table 3). A statistically significant relationship was observed between the subscales of the MHT with the mentioned well-being, mental health, and mental disorder symptoms measures. The absolute values of the correlations ranged between 0.19 and 0.79, with most showing a moderate to very strong level of association. One exception was the correlation between positive experience (%) and self-regulation (r = 0.10), where the relationship was not significant (p > 0.05). Overall, it can be concluded that the MHT subscales perform well with other indicators related to mental health and mental disorders in a clinical sample.

**Table 5:** Correlation of subscales with other well-being scales and measures.

Subscale	Global well-being	Savoring	Creative and executive efficiency	Self-regulation	Psychological resilience	MHT score	N
PERMA-profiler	0.78* (0.73 to 0.82)	0.64* (0.57 to 0.71)	0.59* (0.51 to 0.66)	0.22* (0.11 to 0.32)	0.61* (0.53 to 0.67)	0.81* (0.77 to 0.85)	309
Psychological well-being scale	0.67* (0.61 to 0.73)	0.57* (0.49 to 0.64)	0.59* (0.51 to 0.66)	0.19* (0.08 to 0.29)	0.53* (0.45 to 0.61)	0.73* (0.68 to 0.78)	321
Positive experience %	0.63* (0.56 to 0.69)	0.45* (0.36 to 0.54)	0.33* (0.23 to 0.42)	0.10 (− 0.01 to 0.21)	0.34* (0.25 to 0.44)	0.53* (0.45 to 0.61)	329
Global well-being scale	0.72* (0.66 to 0.77)	0.57* (0.49 to 0.64)	0.59* (0.52 to 0.66)	0.21* (0.09 to 0.31)	0.58* (0.49 to 0.65)	0.76* (0.71 to 0.81)	305
Satisfaction with life scale	0.66* (0.59 to 0.72)	0.49* (0.40 to 0.57)	0.46* (0.37 to 0.54)	0.21* (0.10 to 0.31)	0.50* (0.41 to 0.58)	0.66* (0.59 to 0.72)	322
Positivity scale	0.68* (0.62 to 0.74)	0.57* (0.49 to 0.64)	0.49* (0.41 to 0.58)	0.23* (0.12 to 0.33)	0.53* (0.45 to 0.61)	0.72* (0.67 to 0.77)	316
Symptom Checklist-90	− 0.51* (− 0.60 to − 0.41)	− 0.26* (− 0.37 to − 0.13)	− 0.29* (− 0.41 to − 0.17)	− 0.53* (− 0.61 to − 0.43)	− 0.49* (− 0.59 to − 0.39)	− 0.60* (− 0.68 to − 0.51)	235

N varies due to pairwise selection. Parentheses show confidence intervals (95%) of the presented Pearson correlation coefficients. We compared our results to robust rank-based correlation coefficients (Spearman), with a maximum difference of 0.1 in the absolute strength of correlation (results available from the authors). MHT score is the average of the subscales.

*p < 0.001.

### Results with sociodemographic indicators

Turning to the practical use of the MHT, we first conducted tests to examine whether the MHT is related to any sociodemographic indicators (see Table 6). Our results suggest that in the clinical sample, the overall MHT score, defined by the average of the scores for the five MHT subscales, is not related to the patient’s gender, age, relationship status, number of children, religiosity, or work-related absence. A difference is present in the case of employment (*p* = 0.03), as employed individuals have a higher mean MHT score (3.41) compared to those not in employment (3.21). In terms of educational level, the mean difference between individuals with primary (2.97) and tertiary (3.45) education is significant (*p* = 0.001).

**Table 6:** Difference in MHT scores by sociodemographic indicator.

Variable	Values	Test statistic	*p*-value	N
Gender				
Male	3.35 (0.85)	0.66^a^	0.513	140 (42.7%)
Female	3.29 (0.84)			188 (57.3%)
Age	0.01 (− 0.1 to 0.1)	0.14^b^	0.886	327
Relationship				
Single	3.21 (0.83)	2.84^c^	0.06	163 (49.4%)
Partnered	3.41 (0.86)			149 (45.2%)
Widowed	3.55 (0.66)			18 (5.4%)
Children				
None	3.29 (0.83)	0.46^c^	0.634	179 (54.2%)
1 child	3.41 (0.90)			60 (18.2%)
2 or more children	3.31 (0.83)			91 (27.6%)
Religiosity				
Religious	3.39 (0.74)	0.89^c^	0.413	59 (17.9%)
Not religious	3.36 (0.85)			95 (28.9%)
Own way	3.25 (0.86)			175 (53.2%)
Employment				
Employed	3.41 (0.80)	2.17^a^	0.030	160 (49.7%)
Not employed	3.21 (0.86)			162 (50.3%)
Level of education				
Primary	2.97 (0.79)			46 (14.1%)
Secondary	3.26 (0.80)	7.19^c^	0.001	154 (47.2%)
Tertiary	3.45 (0.80)			126 (38.7%)
Absence from work				
0 days	3.41 (0.84)	0.84^c^	0.434	100 (39.7%)
1–30 days	3.32 (0.86)			121 (48.0%)
31+ days	3.20 (0.71)			31 (12.3%)

^a^Results are from robust t-tests, as the MHT scores were normally distributed by groups, as confirmed by the Shapiro–Wilk test. Values indicate means, with standard deviations in parentheses.

^b^Results are from Pearson correlations. Values indicate the correlation coefficient, with the 95% confidence interval in parentheses. We compared our results to robust rank-based correlation coefficients (Spearman), with a difference of 0.01 in the absolute strength of the correlation (results available from the authors).

^c^For the comparison of multiple means, we used one-way ANOVA tests. Values indicate means, with standard deviations in parentheses. Homogeneity of variances was assumed as Levene’s test was not significant. MHT score is the average of the subscales.

In contrast to sociodemographic indicators, the MHT score is associated with a wide variety of indicators related to bodily symptoms and physical health (see Table 7). The correlations imply that a higher MHT score is significantly related to the lower prevalence of weakness (*r* =  − 0.33), dizziness (*r* =  − 0.19), tiredness (*r* =  − 0.33), nausea (*r* =  − 0.20), headaches (*r* =  − 0.14), fainting (*r* =  − 0.14), muteness (*r* =  − 0.16), loss of sensation (*r* =  − 0.19), and amnesia (*r* =  − 0.29), and higher subjective health (*r* = 0.39), general physical health (*r* = 0.44), and well-being (*r* = 0.44).

**Table 7:** Correlations of MHT score with reported bodily symptoms and physical well-being.

Variable	Values	p-value	N	N and % suffering from symptoms
Backache	− 0.09 (− 0.20 to 0.02)	0.096^a^	330	188 (56.9%)
Weakness	− 0.33 (− 0.43 to − 0.22)	< 0.001^a^	330	250 (75.8%)
Dizziness	− 0.19 (− 0.29 to − 0.08)	0.003^a^	330	176 (53.3%)
Tiredness	− 0.33 (− 0.43 to − 0.23)	< 0.001^a^	330	283 (85.8%)
Nausea	− 0.20 (− 0.30 to − 0.09)	< 0.001^a^	330	97 (29.4%)
Headaches	− 0.14 (− 0.25 to − 0.03)	0.015^a^	330	180 (54.5%)
High blood pressure	− 0.09 (− 0.21 to 0.03)	0.089^a^	330	121 (36.7%)
Fainting	− 0.14 (− 0.24 to − 0.02)	0.005^a^	330	58 (17.6%)
Mutism	− 0.16 (− 0.26 to − 0.06)	0.004^a^	330	63 (19.1%)
Loss of sensation	− 0.19 (− 0.31 to − 0.09)	< 0.001^a^	330	127 (38.5%)
Blindness	− 0.03 (− 0.14 to 0.08)	0.680^a^	330	51 (15.5%)
Convulsions	− 0.05 (− 0.16 to 0.05)	0.355^a^	330	86 (26.1%)
Amnesia	− 0.29 (− 0.39 to − 0.19)	< 0.001^a^	330	173 (52.4%)
Deafness	− 0.07 (− 0.18 to 0.05)	0.202^a^	330	75 (22.7%)
General subj. health	0.39 (0.29 to 0.49)	< 0.001^a^	329	
General phys. health	0.44 (0.35 to 0.53)	< 0.001^a^	329	
General phys. w-b	0.44 (0.34 to 0.53)	< 0.001^a^	329	

^a^Results are from Spearman’s robust rank-based correlation, as normality was violated in most symptom-specific variables and the difference between the Pearson’s and Spearman’s correlation values were major. Values indicate the correlation coefficient, with the 95% bootstrapped (10,000 runs) confidence interval in parentheses. For symptom-specific variables, high scores indicate worse states, while for the general health and well-being variables, higher values indicate better health. MHT score is the average of the subscales.

The MHT scores also perform well in association with mental disorders and symptoms (see Table 8). The average MHT score among those with a self-reported (3.14 compared to 3.76) or diagnosed (3.18 versus 3.54) mental disorder is significantly lower, and a higher number (*r* =  − 0.34, *p* < 0.001) and greater severity (*r* =  − 0.15, *p* = 0.025) of self-reported mental disorders are related to lower overall mental health. Additionally, significant correlations imply that those with higher MHT scores in the clinical sample experienced a lower prevalence of worrying (*r* =  − 0.32), nervousness (*r* =  − 0.41), stress (*r* =  − 0.37), and restlessness (*r* =  − 0.32), and enjoyed better general mental health (*r* = 0.58).

**Table 8:** Association between MHT score and mental disorders and symptoms.

Variable	Values	Test statistic	*p*-value	N
Mental disorder (self-reported)				
Yes	3.14 (0.78)	− 6.00^a^	< 0.001	235 (72.9%)
No	3.76 (0.83)			87 (27.1%)
Mental disorder diagnosis (reported by a professional)				
Yes	3.18 (0.84)	− 3.74^a^	< 0.001	184 (58.9%)
No	3.54 (0.81)			128 (41.1%)
No. of mental disorders (self-reported	− 0.34 (− 0.42 to − 0.22)	− 6.24^b^	< 0.001	331
Average severity of mental disorder(s) (if present) (self-reported)	− 0.15 (− 0.28 to − 0.02)	− 2.15^b^	0.025	220
Worrying	− 0.32 (− 0.42 to − 0.21)	− 6.01^b^	< 0.001	328
Nervousness	− 0.41 (− 0.49 to − 0.31)	− 7.99^b^	< 0.001	328
Stress	− 0.37 (− 0.47 to − 0.27)	− 7.23^b^	< 0.001	328
Restlessness	− 0.32 (− 0.42 to − 0.22)	− 6.14^b^	< 0.001	328
General mental health	0.58 (0.49 to 0.66)	12.79^b^	< 0.001	328

^a^Results are from robust t-tests, as the MHT scores were normally distributed by groups, as confirmed by the Shapiro–Wilk test. Values indicate means, with standard deviations in parentheses.

^b^Results are from Spearman’s robust rank-based correlation, as normality was violated and the difference between the Pearson’s and Spearman’s correlation values were major. Values indicate the correlation coefficient, with the 95% bootstrapped (10,000 runs) confidence interval in parentheses. For symptom-specific variables, high scores indicate worse states, while for the general health and well-being variables, higher values indicate better health. MHT score is the average of the subscales.

### Relationship between psychopathological characteristics and mental health capacities

In the final part of our analysis, we departed from bivariate analyses and fitted multivariate linear (OLS) regressions to investigate the relationship of disorder type, severity of principal diagnosis, psychotherapy during patient care, pharmacotherapy during patient care, and number of self-reported mental disorders with the MHT and its subscales in the presence of socioeconomic controls (see Table 9; the intercorrelations of the SCL-90-R subscales are shown in Supplementary Table 6﻿). Significant associations were found between overall MHT score and unipolar depression and number of mental disorders. Compared to patients with addictive disorders, the MHT score of patients with unipolar depression was 0.44 points lower. Moreover, each additional self-reported mental disorder lowered the MHT score by 0.17 points.

**Table 9:** Linear regression model of MHT score and subscales (base models).

Variable	MHT score	Global well-being	Savoring	Creative and exec. eff	Self-regulation	Psychological resilience
Type (ref.: addictive disorders)						
Affective disorders	− 0.21 (0.19) [− 0.09]	0.11 (0.26) [0.04]	− 0.27 (0.29) [− 0.08]	− 0.26 (0.28) [− 0.09]	− 0.26 (0.28) [− 0.08]	− 0.38 (0.25) [− 0.12]
Anxiety and som. disorders	− 0.34 (0.20) [− 0.12]	− 0.38 (0.28) [− 0.09]	− 0.28 (0.32) [− 0.07]	− 0.17 (0.31) [− 0.04]	− 0.30 (0.31) [− 0.07]	− 0.57* (0.28) [− 0.15]
Personality disorders	− 0.28 (0.19) [− 0.11]	− 0.62* (0.28) [− 0.18]	− 0.32 (0.31) [− 0.09]	− 0.11 (0.29) [− 0.03]	0.00 (0.31) [0.01]	− 0.35 (0.27) [− 0.10]
Psychotic disorders	− 0.05 (0.17) [− 0.03]	− 0.06 (0.24) [− 0.02]	− 0.09 (0.27) [− 0.03]	− 0.46 (0.25) [− 0.17]	0.37 (0.26) [0.13]	− 0.01 (0.23) [− 0.01]
Unipolar depression	− 0.44* (0.19) [− 0.19]	− 0.56* (0.26) [− 0.17]	− 0.78** (0.30) [− 0.23]	− 0.52 (0.28) [− 0.17]	0.49 (0.29) [0.15]	− 0.84** (0.26) [− 0.27]
Severity of principal diag. (ref.: Mild)						
Moderate	− 0.20 (0.18) [− 0.12]	− 0.52* (0.25) [− 0.22]	− 0.22 (0.28) [− 0.09]	− 0.35 (0.27) [− 0.15]	0.23 (0.27) [0.09]	− 0.16 (0.24) [− 0.07]
Severe	− 0.43* (0.18) [− 0.26]	− 0.89* (0.25) [− 0.37]	− 0.45 (0.25) [− 0.18]	− 0.59* (0.27) [− 0.25]	0.24 (0.28) [0.09]	− 0.47 (0.25) [− 0.20]
Psychotherapy (ref.: psychotherapy)						
No psychotherapy	0.15 (0.16) [0.08]	0.04 (0.22) [0.02]	0.43 (0.30) [0.15]	0.07 (0.23) [0.02]	− 0.15 (0.24) [− 0.05]	0.34 (0.21) [0.13]
Pharmacotherapy (ref.: pharmacotherapy)						
No pharmacotherapy	0.32 (0.19) [0.12]	0.24 (0.26) [0.06]	0.47 (0.30) [0.11]	− 0.09 (0.28) [− 0.02]	0.38 (0.29) [0.09]	0.59* (0.26) [0.16]
No. of mental disorders (self-reported)	− 0.17*** (0.04) [− 0.25]	− 0.23*** (0.06) [− 0.24]	− 0.12 (0.07) [− 0.12]	− 0.10 (0.06) [− 0.11]	− 0.17** (0.07) [− 0.18]	− 0.21*** (0.06) [− 0.23]
Gender (ref.: male)						
Female	− 0.12 (0.10) [− 0.07]	− 0.14 (0.14) [− 0.06]	− 0.02 (0.16) [− 0.01]	− 0.14 (0.15) [− 0.06]	0.02 (0.16) [0.01]	− 0.31* (0.14) [− 0.14]
Age	0.00 (0.01) [0.01]	− 0.01 (0.01) [− 0.09]	0.00 (0.01) [− 0.03]	0.00 (0.01) [− 0.03]	0.01 (0.01) [0.03]	0.00 (0.01) [0.04]
Relationship (ref.: single)						
Partnered	0.20 (0.10) [0.12]	0.29* (0.14) [0.12]	0.30 (0.16) [0.12]	0.08 (0.15) [0.03]	0.05 (0.16) [0.02]	0.25 (0.14) [0.11]
Widowed	0.24 (0.28) [0.06]	− 0.01 (0.39) [− 0.00]	0.13 (0.44) [0.02]	− 0.15 (0.41) [− 0.03]	0.66 (0.43) [0.11]	0.57 (0.38) [0.09]
Intercept	3.82*** (0.29)	4.23*** (0.40)	3.99*** (0.46)	4.62*** (0.43)	2.93*** (0.44)	3.33*** (0.39)
Adjusted R^2^	0.14	0.19	0.05	0.02	0.08	0.18
F-statistic	3.93***	5.09***	1.98*	1.35	2.49**	4.89***

(Plots available on request). Parentheses contain the standard errors. Square brackets indicate the standardized regression coefficients. MHT score is the average of the subscales.

N = 251. ***p < 0.001;**p < 0.01;*p < 0.05. The models are acceptable under the assumptions of linear regression, except the model for creative and executive efficiency, where the global test of significance indicated that the model does not fit.

Models for the subscales yielded additional insights. Compared to the reference group (addictive disorders), those with personality disorders (*b* =  − 0.62) and unipolar depression (*b* =  − 0.56) had lower global well-being scores. Severity was also a key factor for global well-being, compared to a mild principal diagnosis: those with moderate (*b* =  − 0.52) and severe (*b* =  − 0.89) diagnoses had lower scores. The effect of the number of self-reported mental disorders (*b* =  − 0.23) was similar to that of the overall MHT. In the case of savoring, the only significant association was found with unipolar depression (*b* =  − 0.78), which is connected with reduced savoring capacity. Similarly, only one variable—number of self-reported mental disorders—was significantly negatively associated with self-regulation (*b* =  − 0.17). Finally, the psychological resilience score was negatively associated with anxiety and somatization disorders (*b* =  − 0.57), unipolar depression (*b* =  − 0.84), and number of self-reported mental disorders (*b* =  − 0.27), but positively related to pharmacotherapy (*b* = 0.59). The model for creative and executive efficiency did not fit our data.

In addition to the main models presented above, we fitted additional models with an alternative psychotherapy and pharmacotherapy specification (see Table 10). As other effects were unchanged, they are not presented again. The results show that combinations of psychotherapy and pharmacotherapy are positively related to the overall MHT, and to the creative and executive efficiency and psychological resilience subscales. Compared to patients receiving neither psychotherapy nor pharmacotherapy during their care, those receiving psychotherapy but not pharmacotherapy had a significantly higher MHT score (*b* = 0.87). For the creative and executive efficiency subscale, the combinations are especially important: receiving therapy without medication (*b* = 1.96), medication only (*b* = 1.81), or a combination (*b* = 1.59) significantly improved score. For psychological resilience, only therapy without medication was found to be a significantly positive factor (*b* = 1.46).

**Table 10:** Linear regression model of MHT score and subscales with psychotherapy–pharmacotherapy combinations.

Variable	MHT score	Well-being	Savoring	Creative and exec. eff	Self-regulation	Psychological resilience
Pharmacotherapy–Psychotherapy (ref: psychotherapy and pharmacotherapy)						
Psychotherapy with no pharmacotherapy	0.55* (0.21) [0.19]	0.45 (0.29) [0.11]	0.73* (0.34) [0.17]	0.37 (0.31) [0.09]	0.18 (0.33) [0.04]	0.99** (0.0.28) [0.05]
Pharmacotherapy with no psychotherapy	0.22 (0.16) [0.12]	0.11 (0.22) [0.04]	0.52* (0.25) [0.18]	0.21 (0.23) [0.08]	− 0.21 (0.25) [− 0.08]	0.47* (0.21) [0.18]
No psychotherapy and no pharmacotherapy	− 0.32 (0.42) [− 0.05]	− 0.47 (0.59) [− 0.05]	0.03 (0.67) [0.01]	− 1.59** (0.62) [− 0.17]	0.88 (0.65) [0.08]	− 0.46 (0.57) [− 0.05]
Adjusted R^2^	0.16	0.19	0.06	0.06	0.08	0.21
F-statistic	4.15***	4.97***	2.07*	2.05*	2.45**	5.45***

(Plots available on request). The models control for all variables listed in the models in Table 8. Parentheses contain the standard errors. Square brackets indicate the standardized regression coefficients. MHT score is the average of the subscales.

N = 251. ***p < 0.001;**p < 0.01;*p < 0.05. The models are acceptable under the assumptions of linear regression.

## Discussion

The present study aims to examine the reliability and validity of the MHT in a Hungarian adult psychiatric sample and the relationship between mental health competencies and various indicators (sociodemographic characteristics, physical health, mental disorders, psychotherapy and/or pharmacotherapy during care). Confirmatory factor analysis showed a good fit of the five-factor model to the data for the clinical version of the Mental Health Test. High internal consistency coefficients and excellent external and content validity were reported. The test is not sensitive to sociodemographic indicators but is sensitive to the correlates of well-being and to the symptoms of different types of mental disorders. Our findings suggest that the Mental Health Test is a suitable measure for assessing mental health capacities in psychiatric samples.

The test is innovative in several ways, particularly in the clinical setting, compared to previous tests. First, it is based on the Maintainable Positive Mental Health Theory (MPMHT). One of the key messages of this concept is that there are various competencies behind the different components of mental health unlike previous measures which define mental health in terms of observable characteristics of well-being or as a mirror opposite of mental disorders. The MPMHT captures not just one (e.g. resilience^9^, social well-being^40^, coping^41^, but all of the essential aspects of mental health as defined by the World Health Organization^12^ and classical mental health theories. The identified main pillars of positive mental health—global well-being, creative and efficient coping, savoring capacity, resilience, and dynamic self-regulation—are competencies that can be trained, improved, and strengthened by their nature. This multidimensional approach, that channels five scales into a comprehensive framework shows conceptual similarities with recent transformative mental health models e.g. pivotal mental states model^42^. The MHT covers a wide spectrum of mental health measures and suitable for the comprehensive assessment of the individual's mental health competencies. The short completion time, the self-test design can provide the opportunity for a quick-and-easy assessment even in time-pressured clinical settings.

Furthermore, on the basis of this assessment, with precisely targeted interventions or even with self-help activity, people living with mental disorder(s) can establish a balance between their own physical and mental status and their social environment, and they can also create a sustainable optimization of personal and social functioning (self-regulation) and an equilibrium of positive and negative emotions (coping, savoring). It may also increase the level of spiritual connectedness, sense of coherence^43^, and ultimately global functioning^14^. This shift in perspective aligns with the growing recognition of the importance of promoting positive mental health and well-being (Supplementary Fig. 1).

### Limitations

Firstly, the validation was carried out on a convenience sample, thus the resulting sample is not representative of patients in the participating healthcare facilities. Furthermore, measurement invariance could not be tested due to the small sample size in the subsamples. Like all self-report questionnaires, the MHT is, to a certain extent, liable to the conscious and unconscious response tendencies of the respondents. In addition, since participation was voluntary, more severe cases were not represented, while various factors related to personality, illness, and attitude, as well as factors related to mental well-being, are likely to have influenced willingness to participate. Additionally, the intercorrelations of the MHT subscales are very high, which is due to the fact that the scales of the MHT are inspired by previous measures (e.g. the Global Well-being subscale was derived from the Global Well-being Scale^15^). Despite that, the advantage of the instrument is the comprehensiveness that results from its multidimensionality. It measures five mental health competencies and an average indicator which provides a more individualistic insight into the functioning of a person’s mental health competencies. Another limitation is that the measure shows strong positive correlation with other well-being and mental health scales and instrument. This may result from the fact that quantitative scales cannot capture such subtle differences in a person’s mental health characteristics, which can be crucial in therapeutic practice, for example. Finally, in the analysis of the interrelationships between combinations of psychotherapy and pharmacotherapy and mental health, it would be worth filtering out the covariate effect of type of care.

### Conclusion and consequences

In summary, our preliminary results suggest that the MHT is a suitable measure for assessing mental health competencies and resources in psychiatric samples. Exploring the positive dimensions of people living with mental disorders is not only of theoretical importance but also has purposeful practical consequences. Firstly, it reveals the foundations on which a rehabilitation professional group can build in order to achieve positive life goals (including destigmatization and reducing self-stigma), where the goal is not necessarily symptom reduction but improving quality of life and the restoration of everyday functionality to the fullest extent possible. Secondly, and as a result of the above, it can propose a new paradigm for therapy which not only focuses on treating symptoms and maximizing adaptation level, but also puts a huge emphasis on the empowerment of the patients. Thirdly, it may have health-related and economic significance, since people living with mental disorders may be helped to recover their functions more quickly, enabling them to return to social productivity sooner. It can also provide an opportunity to embrace disability as part of human experience and to support patients based on their existing psychological competencies.

### Supplementary Information

Supplementary Information.

## Data Availability

The datasets generated and/or analysed during the present study are available from the corresponding author on reasonable request.
