# Supplementary material for: Preliminary validation of the Mental Health Test in a psychiatric sample
Source: Sci Rep. 2024 Feb 18;14:4023. doi: 10.1038/s41598-024-54537-4 (PMC10874926; doi:10.1038/s41598-024-54537-4)
Supplement: Supplementary file 1 — Supplementary Information. [file 41598_2024_54537_MOESM1_ESM.docx]

1. **Supplementary Material**

**Figure 1. Density plots of the scales**


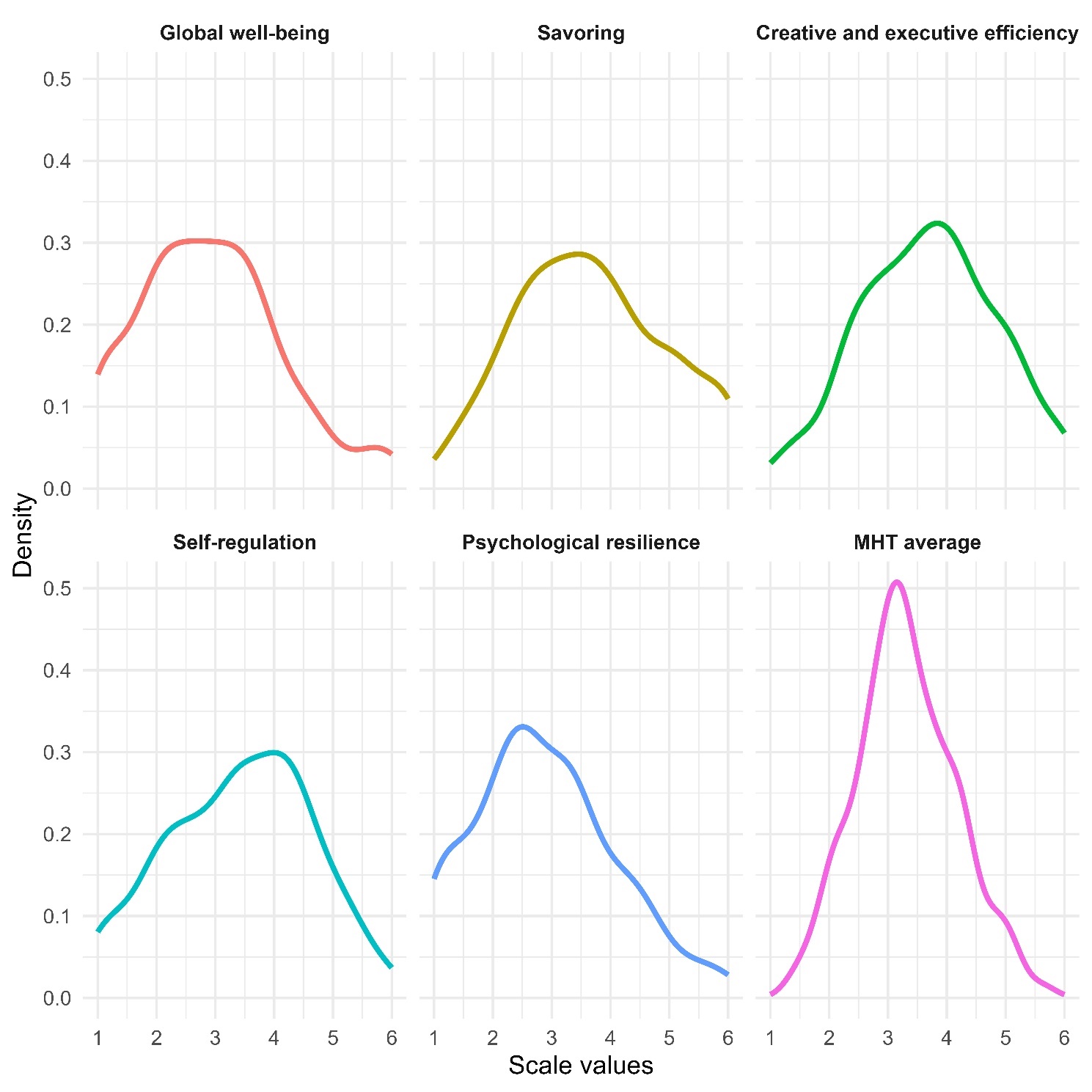


**Table 1. Details of measures**

| **Name of the measure** | **Description of the measure** |
| --- | --- |
| Mental Health Test^14^ | The 17-item self-report test operationalises the Maintainable Positive Mental Health Theory^13,14^ and measures five mental health capacities and competencies in adult population: Global Well-being, Savoring, Resilience, Self-regulation, and Creative and Executive Efficiency. Respondents rate 17 items on a 7-point Likert scale from 1 (strongly disagree) to 7 (strongly agree). The MHT score is defined as the average of the scores for the five subscales, which provide a comprehensive picture of the respondent’s level of mental health capacities. The detailed instructions for the scaling guide are given in Table 2 in the Appendices. |
| Global Well-being Scale^15^ | This self-report questionnaire operationalises the bio-psycho-socio-spiritual model of well-being, which emphasises that the condition for complete well-being is to function well in the emotional, psychological, social and spiritual domains of one's human nature. The 17 statements in the questionnaire are rated on a 7-point Likert scale from 1 (strongly disagree) to 7 (strongly agree). |
| PERMA-profiler^27^ | The five-pillar model measures positive emotions, engagement, positive relationship, meaning, accomplishment which reinforce one another in creating and maintaining a state of well-being. The self-report 23-items are rated on a 10-point Likert scale from 1 (strongly disagree) to 10 (strongly agree). |
| Psychological Well-being Scale^3^ | This widely used self-report scale operationalises an improved version of Diener's concept of subjective well-being, which emphasises the need for competence, optimism, contribution to the well-being of others, purpose in life, self-esteem, and positive relationships, in addition to life satisfaction and the dominance of positive emotions. The 8 statements in the questionnaire are rated on a 7-point Likert scale from 1 (strongly disagree) to 7 (strongly agree). |
| Satisfaction with Life Scale^28^ | This self-report scale measures overall life satisfaction by rating five items on a seven-point Likert scale from 1 (strongly disagree) to 7 (strongly agree). |
| Positivity Scale^6^ | Respondents rate the eight items on a 5-point scale from 1 (strongly disagree) to 5 (strongly agree) that measure the individual's tendency to view their life and experiences in a positive way. |
| Symptom Checklist-90, revised^29^ | This self-report inventory measures the presence and degree of severity of psychological symptoms on 9 scales: somatization, compulsivity, interpersonal sensitivity, depression, anxiety, hostility, phobia, paranoia, psychoticism, and a group of additional items not included in any of the scales. The Global Severity Index, in contrast to the problem-specific scales, provides an overall picture of the general severity of mental disorder symptoms. The 90 statements in the questionnaire are rated on a 5-point Likert scale. |

**Table 2. Descriptive statistics of the other well-being scales in the study**

| **Subscale** | **Mean** | **Median** | **SD** | **Skewness** | **Kurtosis** | **Range** | **N** |
| --- | --- | --- | --- | --- | --- | --- | --- |
| PERMA-profiler | 5.59 | 5.52 | 1.64 | 0.12 | -0.44 | 1.71–9.57 | 309 |
| Psychological Well-being Scale | 4.25 | 4.25 | 1.47 | -0.06 | -0.72 | 1–7 | 321 |
| Positive Experience % | 5.07 | 5.00 | 2.68 | 0.10 | -1.16 | 1–10 | 329 |
| Global Well-being Scale | 3.19 | 3.11 | 1.22 | 0.2 | -0.61 | 1–6 | 305 |
| Satisfaction with Life Scale | 3.42 | 3.20 | 1.55 | 0.30 | -0.87 | 1–7 | 322 |
| Positivity Scale | 3.12 | 3.12 | 0.87 | 0.01 | -0.77 | 1–5 | 316 |
| Symptom Checklist-90 | 1.12 | 1.02 | 0.69 | 0.61 | -0.12 | 0.01–3.15 | 235 |

**Table 3. Measures of reliability for the other well-being scales and measures**

| **Subscale** | **Number of items / subscales** | **Cronbach’s α** | **McDonald’s ω** | **N** |
| --- | --- | --- | --- | --- |
| PERMA-profiler | 7 | 0.86 (0.83–0.88) | 0.87 (0.85–0.89) | 309 |
| Psychological Well-being Scale | 8 | 0.91  (0.89–0.93) | 0.91 (0.89–0.93) | 321 |
| Positive Experience % | 1 | - | - | 329 |
| Global Well-being Scale | 4 | 0.91 (0.89–0.93) | 0.91 (0.89–0.93) | 305 |
| Satisfaction with Life Scale | 5 | 0.87 (0.84–0.89) | 0.87 (0.84–0.89) | 322 |
| Positivity Scale | 8 | 0.83 (0.81–0.86) | 0.86 (0.84–0.88) | 316 |
| Symptom Checklist-90 | 9 | 0.93 (0.92–0.95) | 0.94 (0.92–0.95) | 235 |

**Table 4. Descriptive statistics of the MHT subscales (n = 331)**

| **Subscale** | **Mean** | **Median** | **SD** | **Skewness** | **Kurtosis** | **Range** |
| --- | --- | --- | --- | --- | --- | --- |
| Global well-being | 2.91 | 3.00 | 1.21 | 0.45 | -0.21 | 1–6 |
| Savoring | 3.67 | 3.67 | 1.26 | 0.14 | -0.79 | 1–6 |
| Creative and executive efficiency | 3.69 | 3.80 | 1.15 | -0.04 | -0.56 | 1–6 |
| Self-regulation | 3.41 | 3.33 | 1.22 | -0.12 | -0.72 | 1–6 |
| Psychological resilience | 2.89 | 2.67 | 1.18 | 0.41 | -0.34 | 1–6 |
| MHT score | 3.32 | 3.25 | 0.84 | 0.22 | -0.22 | 1.35–5.67 |

**Table 5. Descriptive statistics of the single items of the MHT scale (n = 331)**

| **Item** | **Mean** | **Median** | **SD** | **Skewness** | **Kurtosis** | **Range** | **Cronbach’s α of the given subscale if item is deleted** |
| --- | --- | --- | --- | --- | --- | --- | --- |
| 01W | 2.95 | 3.00 | 1.43 | 0.46 | -0.53 | 1–6 | 0.82 (0.77–0.87) |
| 13W | 2.80 | 3.00 | 1.42 | 0.56 | -0.39 | 1–6 | 0.76 (0.68–0.83) |
| 17W | 3.01 | 3.00 | 1.31 | 0.23 | -0.49 | 1–6 | 0.74 (0.66–0.80) |
| 03S | 3.74 | 4.00 | 1.51 | 0.05 | -1.02 | 1–6 | 0.64 (0.54–.72) |
| 09S | 3.95 | 4.00 | 1.53 | -0.20 | -0.98 | 1–6 | 0.68 (0.59–0.75) |
| 11S | 3.31 | 3.00 | 1.57 | 0.20 | -0.93 | 1–6 | 0.72 (0.63–0.79) |
| 05C | 3.50 | 4.00 | 1.52 | -0.05 | -0.82 | 1–6 | 0.79 (0.74–0.83) |
| 06C | 3.66 | 4.00 | 1.51 | -0.10 | -0.87 | 1–6 | 0.78 (0.72–0.82) |
| 08C | 3.51 | 4.00 | 1.41 | -0.06 | -0.58 | 1–6 | 0.82 (0.77–0.85) |
| 14C | 3.74 | 4.00 | 1.52 | -0.05 | -0.94 | 1–6 | 0.82 (0.78–0.85) |
| 16C | 4.06 | 4.00 | 1.34 | -0.27 | -0.55 | 1–6 | 0.84 (0.81–0.87) |
| 02SR | 3.38 | 3.00 | 1.51 | -0.05 | -0.95 | 1–6 | 0.57 (0.45–0.66) |
| 07SR | 3.81 | 4.00 | 1.62 | -0.33 | -1.00 | 1–6 | 0.63 (0.53–0.72) |
| 15SR | 3.97 | 4.00 | 1.47 | -0.19 | -0.90 | 1–6 | 0.64 (0.54–0.71) |
| 04PR | 3.01 | 3.00 | 1.44 | 0.43 | -0.56 | 1–6 | 0.68 (0.59–0.76) |
| 10PR | 2.77 | 3.00 | 1.45 | 0.64 | -0.36 | 1–6 | 0.59 (0.47–0.68) |
| 12PR | 2.88 | 3.00 | 1.35 | 0.28 | -0.61 | 1–6 | 0.83 (0.78–0.87) |

*Note.* The numbering of the items correspond to the questions presented in table 2 of the supplementary material. W = Global well-being; S = Savoring; C = Creative and executive efficiency; SR = Self-regulation; PR = Psychological resilience.

**Table 6. Correlation of the Symptom Checklist-90 subscales in the sample (n = 235)**

| **Subscale** | **Somatization** | **Obsessive-Compulsive** | **Interpersonal Sensitivity** | **Depression** | **Anxiety** | **Hostility** | **Phobic Anxiety** | **Paranoid Ideation** | **Psychotism** |
| --- | --- | --- | --- | --- | --- | --- | --- | --- | --- |
| **Somatization** |  | 0.46** (0.33–0.57) | 0.45** (0.34–0.56) | 0.58** (0.49–0.67) | 0.68** (0.60–0.75) | 0.42** (0.28–0.55) | 0.50** (0.39–0.61) | 0.43** (0.29–0.55) | 0.48** (0.33–0.58) |
| **Obsessive-Compulsive** |  |  | 0.72** (0.66–0.78) | 0.75** (0.59–0.80) | 0.76** (0.69–0.82) | 0.53** (0.44–0.61) | 0.59** (0.50–0.69) | 0.61** (0.51–0.69) | 0.72** (0.64–0.79) |
| **Interpersonal Sensitivity** |  |  |  | 0.73** (0.68–0.79) | 0.74** (0.68–0.79) | 0.60** (0.50–0.69) | 0.56** (0.47–0.65) | 0.77** (0.71–0.83) | 0.74** (0.67–0.80) |
| **Depression** |  |  |  |  | 0.80** (0.75–0.85) | 0.49** (0.40–0.59) | 0.52** (0.42–0.60) | 0.60** (0.51–0.68) | 0.65** (0.57–0.71) |
| **Anxiety** |  |  |  |  |  | 0.64** (0.56–0.72) | 0.71** (0.64–0.77) | 0.66** (0.58–0.74) | 0.68** (0.61–0.75) |
| **Hostility** |  |  |  |  |  |  | 0.46** (0.36–0.56) | 0.62** (0.53–0.70) | 0.56** (0.46–0.65) |
| **Phobic Anxiety** |  |  |  |  |  |  |  | 0.54** (0.44–0.64) | 0.51** (0.41–0.61) |
| **Paranoid Ideation** |  |  |  |  |  |  |  |  | 0.70** (0.63–0.77) |
| **Psychotism** |  |  |  |  |  |  |  |  |  |

Results are from Spearman’s robust rank-based correlation, as normality was violated and the difference between the Pearson’s and Spearman’s correlation values were major. Values indicate the correlation coefficient, with the 95% bootstrapped (10,000 runs) confidence interval in parentheses.

****p* < 0.001; **p < 0.01; **p* < 0.05.

**Table 7. The Mental Health Test (MHT)**

The following statements are designed to obtain information about your perceptions of wellness. Please consider each statement carefully and thoughtfully, then enter an X to indicate the response option with which you most agree. There are no right or wrong answers.

|  | Strongly disagree | Disagree | Slightly disagree | Slightly agree | Agree | Strongly agree |
| --- | --- | --- | --- | --- | --- | --- |
| 1. Joy is present more than sorrow in my daily life. (W) |  |  |  |  |  |  |
| 2. I easily become impatient. (SR) |  |  |  |  |  |  |
| 3. It’s easy for me to revive my joy from pleasant memories. (S) |  |  |  |  |  |  |
| 4. I tend to bounce back quickly after hard times. (PR) |  |  |  |  |  |  |
| 5. I often have ideas that are taken further by others. (C) |  |  |  |  |  |  |
| 6. Others describe me as a problem solver. (C) |  |  |  |  |  |  |
| 7. I am impulsive: I act first and think second. (SR) |  |  |  |  |  |  |
| 8. I can successfully achieve targets which I set for myself. (C) |  |  |  |  |  |  |
| 9. I like to store memories of fun times that I go through so that I can recall them later. (S) |  |  |  |  |  |  |
| 10. It does not take me long to recover from a stressful event. (PR) |  |  |  |  |  |  |
| 11. I can make myself feel good by imagining what a happy time that is about to happen will be like. (S) |  |  |  |  |  |  |
| 12. I tend to take a long time to get over setbacks in my life. (PR) |  |  |  |  |  |  |
| 13. My general psychological state is good. (W) |  |  |  |  |  |  |
| 14. I am good at jobs that need new and original ideas. (C) |  |  |  |  |  |  |
| 15. I become frustrated when something does not happen the way I planned it. (SR) |  |  |  |  |  |  |
| 16. I often know how people think and feel. (C) |  |  |  |  |  |  |
| 17. How do you feel about your life as a whole?  (1: Very badly, 6: Very well) (W) |  |  |  |  |  |  |

*Note.* W = Global well-being; S = Savoring; C = Creative and executive efficiency; SR = Self-regulation; PR = Psychological resilience.

Scaling guide:

Well-being: The average of the scores for items 1, 13, and 17

Savoring: The average of the scores for items 3, 9, and 11

Creative and executive efficiency: The average of the scores for items 5, 6, 8, 14, and 16

Self-regulation: The average of the scores for reversed items 2, 7 and 15

Psychological resilience: The average of the scores for items 4 and 10 and the reversed score for item 12

The total score for the Mental Health Test is defined as the average of the scores for the five subscales.

**Table 8. Mentális Egészség Teszt (MET)**

Az alábbi állítások az emberek általános életérzését, jellemző tulajdonságait és életfelfogását írják le. Nincsenek jó vagy rossz válaszok. Kérem, legyen olyan őszinte, amennyire csak tud és jelezze minden tételnél a megfelelő szám bekarikázásával, hogy milyen mértékben jellemző Önre az állítás!

|  | **egyáltalán nem jellemző** | **nem jellem­ző** | **kicsit jellem­ző** | **jel­lemző** | **nagyon jellem­ző** | **teljes mértékben jellemző** |
| --- | --- | --- | --- | --- | --- | --- |
| 1. Mindennapjaimban érezhetően több az öröm, mint a bánat. (J) | 1 | 2 | 3 | 4 | 5 | 6 |
| 2. Könnyen válok türelmetlenné. (Ö) | 1 | 2 | 3 | 4 | 5 | 6 |
| 3. Könnyen fel tudom eleveníteni a múlt kellemes emlékeinek örömét. (S) | 1 | 2 | 3 | 4 | 5 | 6 |
| 4. Nehéz idők után hamar magamhoz térek. (R) | 1 | 2 | 3 | 4 | 5 | 6 |
| 5. Gyakran vannak olyan ötleteim, amelyekhez mások eredményesen tudnak kapcsolódni és továbbgondolkodásra készteti őket. (AV) | 1 | 2 | 3 | 4 | 5 | 6 |
| 6. Mások szerint is jó problémamegoldó vagyok. (AV) | 1 | 2 | 3 | 4 | 5 | 6 |
| 7. Hirtelen természetű vagyok (előbb cselekszem, utána gondolkodom). (Ö) | 1 | 2 | 3 | 4 | 5 | 6 |
| 8. Sikeresen el tudom érni a magam elé kitűzött célokat. (AV) | 1 | 2 | 3 | 4 | 5 | 6 |
| 9. Szeretem elraktározni az átélt örömteli idők emlékét, hogy később felidézhessem őket. (S) | 1 | 2 | 3 | 4 | 5 | 6 |
| 10. A lelki megrázkódtatások után elég gyorsan felépülök. (R) | 1 | 2 | 3 | 4 | 5 | 6 |
| 11. Jó kedvre tudom hangolni magam, ha elképzelem, milyen lesz egy közelgő boldog idő. (S) | 1 | 2 | 3 | 4 | 5 | 6 |
| 12. Rendszerint eltart egy ideig, amíg továbblépek életem egy-egy nehéz pillanatán. (R) | 1 | 2 | 3 | 4 | 5 | 6 |
| 13. Lelki állapotom jónak mondható. (J) | 1 | 2 | 3 | 4 | 5 | 6 |
| 14. Jó vagyok az olyan munkákban, ahol új és eredeti ötletek kellenek. (AV) | 1 | 2 | 3 | 4 | 5 | 6 |
| 15. Ideges leszek, ha valami nem úgy alakul, ahogy terveztem. (Ö) | 1 | 2 | 3 | 4 | 5 | 6 |
| 16. Gyakran jók a megsejtéseim arról, hogy hogyan gondolkoznak és éreznek az emberek. (AV) | 1 | 2 | 3 | 4 | 5 | 6 |
| 17. Mindent összevetve mennyire mondaná magát boldognak (1: nagyon nem, 6: nagyon igen)? (J)  1 2 3 4 5 6 | | | | | | |

Megjegyzés: J = Jóllét; S = Savoring; AV = Alkotó–végrehajtó hatékonyság; Ö = Önreguláció; R = Reziliencia.

Skálaképzési útmutató:

Jóllét (J): 1, 13 és17 tételek pontszámainak átlaga

Savoring (S): 3, 9 és 11 tételek pontszámainak átlaga

Alkotó–végrehajtó hatékonyság (AV): 5, 6, 8, 14 és 16 tételek pontszámainak átlaga

Önreguláció (Ö): 2, 7 és 15 fordított tételek pontszámainak átlaga

Reziliencia (R): 4 és 10 tételek pontszámainak és a 12 fordított tétel pontszámának átlaga

A Mentális Egészség Teszt összpontszáma az öt alskála pontszámainak átlaga.
